# Association of mental health and behavioral disorders with health care and service utilization in children before and after diagnosis

**DOI:** 10.1371/journal.pone.0278198

**Published:** 2022-11-28

**Authors:** Mikko Nurminen

**Affiliations:** The Social Insurance Institution of Finland (Kela), Helsinki, Finland; Rey Juan Carlos University: Universidad Rey Juan Carlos, SPAIN

## Abstract

Mental health is one of the most important contributors to the global burden of disease in children and adolescents. Mental health conditions are associated with lower quality of life in adulthood. These conditions have an early onset and typically first occur in childhood. However, little is known about how these conditions are related to service utilization before the initial diagnosis, or about the significance of the diagnosis on later service utilization. To study this, register data on 5-15-year-old children in the city of Oulu, Finland, covering the years 2013–2018 were used. To identify the association of mental health and behavioral conditions with service utilization, counterfactuals were constructed from children who were similarly diagnosed three years later. Event study regressions on several health care and service utilization outcomes were estimated. The findings showed that primary and specialized health care utilization increased sharply before the initial diagnosis and peaked during the time of diagnosis. Primary care utilization started decreasing slowly after, while specialized health care utilization remained high for two years after the diagnosis. Probability of visiting a mental health professional, use of rehabilitation services, psychiatric medication, and utilization of child protection services increased significantly after the diagnosis. The results highlighted the importance of outpatient health care in detecting and treating the conditions in children. In a fragmented system, knowledge on utilization trajectories in different services may be of help in allocating resources to improve the health of these children.

## Introduction

Mental health is one of the most important contributors to the global burden on disease in children and adolescents [1, 2]. Mental health and behavioral disorders top the list of the costliest conditions [3]. These conditions are also associated with lower educational attainment [4] and disrupted life and functioning [5–8] in adulthood. Mental and behavioral disorders have an early onset and typically first occur in childhood [9, 10]. Preventive treatment could lower the burden of health care resource utilization and control costs in diagnoses such as psychosis [11], while improved mental health during childhood may help with developmental challenges in adulthood. For example, early detection, intervention, and identification of the predictors of psychosis for ultra-high risk patient populations has gained attention in the recent literature [12–15]. However, more research is needed on how mental health and behavioral disorders in children are related to service utilization before the initial diagnosis and the significance of the diagnosis on later service utilization.

In comparison to non-mentally ill children, mental health conditions are associated with higher and recurrent utilization of health care [6, 16–18]. Utilization of psychiatric medication is also higher among these children and has been increasing during the past two decades [19–21]. Additionally, mental health conditions have been associated with higher spending on non-mental health conditions [22]. One reason could be that mental health diagnoses are typically more prevalent in vulnerable populations. For example, the probability of mental health diagnoses in children placed in out-of-home care (foster care) is considerably higher [23–25]. Despite the higher utilization of services, treatment gaps seem to persist in mental health conditions [26].

Previous literature has focused on service utilization before the diagnosis [27, 28] or around a specific diagnosis such as bipolar disorder, binge-eating disorder, or psychosis either without a comparison group, or has constructed the control group from the non-diagnosed population [14, 29–31]. The literature has not explicitly addressed the potential selection bias of the study population. Another strand of literature has focused on service utilization patterns or predictors of service utilization in children enrolled in specific community-based mental health service organizations [32] or in communities that have implemented evidence-based practices (EBPs) [33].

In contrast to the previous literature, I constructed the control group from children who received the same mental health diagnoses but three years later. The idea behind this was to overcome the challenge of selection bias and unobserved heterogeneity [34, 35]. In other words, the idea was to increase the plausibility that the groups were similar to each other also in unobservable factors. A three-year window was chosen so that the outcomes could be tracked for sufficient amount of time and that the treatment and control groups remained similar to each other. A “placebo” diagnosis was then assigned to the control group to take place three years earlier than their actual initial diagnosis. The groups were then compared to each other before and after the diagnosis in a difference-in-differences framework. I leveraged comprehensive register data gathered from various sources on the child population of one Finnish city over the period 2013–2018.

The objectives of this study were as follows. First, to analyze the association of mental health and behavioral conditions with pre-diagnosis health care and service utilization. Second, to analyze how the trajectory of service utilization changed in a two-year follow-up and what was the association of the initial diagnosis. Finally, comparisons of the composition of the pre- and post-diagnosis health care service utilization were made to observe shifts between visits related to mental health and other visits.

The next section presents the Finnish institutional setting, the data, and the empirical approach.

## Materials and methods

### Finnish institutional setting

Finland has a universal health care system [36]. Public health care is financed through taxes and small user fees and co-payments for treatment. Public primary care is organized by municipal health centers. Public specialized health care is provided through 20 hospital districts [36]. Each municipality is part of a hospital district. Physicians and nurses in primary care act as gatekeepers to specialized health care. Hospital districts are also responsible for organizing on-call duty (walk-in visits) services for conditions that require urgent care. On-call duty visits are visits that are made without appointment. Typically, on-call duty visits are made to outpatient setting. However, if urgent specialist examinations or procedures are needed, the patient is referred to the specialized care.

Child health clinics monitor and examine the physical and mental health of children under school age. School health care is a health care service for primary school students, and is typically located in schools or nearby locations. Child health clinic and school health care services are provided free of charge. Municipalities are required to organize regular health checks for children [36]. At least six health checks are required for children aged 1–6 years and at least nine checks are required for children aged under one. Also, health checks must be arranged every school year.

The Finnish private health care sector is market-based. Private health care is mainly financed through out-of-pocket payments and private voluntary health insurance [36]. A small fraction of the out-of-pocket costs are also reimbursed by the national health insurance (NHI) scheme. Finnish residents are entitled to the NHI scheme. Voluntary private health insurance can be purchased on top of the NHI scheme. Apart from sickness insurance for children, voluntary health insurance has not been very common in Finland [36]. In private health care, an appointment with a specialist can be made directly without a referral from primary care. The private sector mostly offers outpatient care.

Rehabilitation services can be provided for mental health and behavioral conditions. In the context of children and adolescents, these services typically include rehabilitation, adaption training courses tailored for specific mental health diagnoses, and rehabilitative psychotherapy. Rehabilitation services are provided by the Social Insurance Institution of Finland along with health centers and hospitals [36]. Most of the statutory services are provided free of charge. Municipalities’ rehabilitative care can often be outsourced to private providers. In this case, they are not part of the NHI scheme.

In severe mental and behavioral disorders, psychiatric medicines can be used as one element of the treatment [37]. Prescription medicines that have been confirmed as reimbursable by the Pharmaceuticals Pricing Board are reimbursed by the NHI. The reimbursement is usually provided directly at the pharmacy. The basic rate of reimbursement is 40% of the retail price.

In Finland, a child may be placed with child protection services if he or she is neglected at home or the child’s own behavior endangers his or her health or development [24]. Municipalities are responsible for organizing child protection services. These services can be given as either in-home or out-of-home care. In-home child protection consists of various guidance, counselling and supportive services. Out-of-home child protection consists of family foster care and institutional care. Out-of-home care is typically a measure of last resort [24].

### Data sources

Data were collected from several registers for the population aged 5–15 of the city of Oulu, Finland, for the period 2013–2018. With an approximate population of 200,000, Oulu is the fifth largest city in Finland. Pseudonymized individual identifiers were used to link data from different registers.

#### Health care services

Public primary care visits were gathered from two separate registers: the register of the city of Oulu and the Register of Primary Health Care Visits, which is maintained by the Finnish Institute for Health and Welfare. These registers contained visits to child health care, school health care, and the regular primary care available at health centers. Information included the date of the visit, specialty of the health care professional, diagnosis codes, and type of service (e.g. school health care, mental health care, and other outpatient care). These two sets of register data covered much of the same ground but also complemented each other in terms of missing information in the variables. For example, in rare cases, one register may contain the diagnosis given during the visit while the other register may not. Also, a visit may include several inconsistently recorded events, which made it unreliable to measure the number of separate visits during one day with the same service provider. For these reasons, I limited the number visits to maximum of one per day per person.

Data on public specialized care visits were gathered from the Care Register of Health Care, maintained by the Finnish Institute for Health and Welfare. This register contained data on inpatient and outpatient specialized health care services. Information in the data included the date of the visit, diagnosis codes, specialty of the health care professional, and service type of the provider. I used the service type to separately identify on-call duty visits. I used it also to identify visits that involved a scheduled appointment and daytime hospital care. I identified distinct visits with separate contact days.

Data on private health care visits came from the registers of the Social Insurance Institution of Finland. These data included visits and procedures reimbursed by the NHI scheme. Additionally, the data record the date of the visit and specialty of the health care professional. Similarly to the above datasets, I identified separate visits using distinct dates.

In all of the aforementioned registers, I separately identified visits to mental health professionals (see S1 File). Specialties included psychiatrists, psychologists, psychotherapists, and occupational therapists.

The number of health care visits (primary care, specialized care, on-call duty, and private care) were measured at child-quarter level. Visits to mental health professionals were measured with a binary variable. The binary variable indicated whether a child visited a mental health professional during a given quarter or not.

#### Mental health diagnosis

In Finland, only physicians can diagnose mental health conditions. Information on mental health diagnoses came from public primary care and public inpatient and outpatient specialized care. The private health care visits were not used as this register did not record diagnoses. Public primary care visits in both the Register of the city of Oulu and the register of Primary Health Care Visits had diagnoses recorded separately in ICPC-2 (The International Classification of Primary Care) coding and ICD10 (The International Classification of Diseases) coding. ICPC-2 is a coding system dedicated to classifying diagnoses in primary care, and, in the public primary care data, ICPC-2 diagnoses were more consistently recorded than ICD10 diagnoses. The specialized health care data used ICD10 coding. In the ICPC-2 coding, I included all primary diagnoses in the “P” category (psychological). In the ICD10 coding, I included all primary diagnoses in the “F” category (mental and behavioral disorders).

#### Rehabilitation

Data on the rehabilitation covered by the NHI were drawn from the registers of the Social Insurance Institution of Finland. The rehabilitation services provided by the Social Insurance Institution include, for example, rehabilitative psychotherapy and intensive medical rehabilitation. The data identified the start and end points of the service. Rehabilitation can be partly embedded in the primary care data, but I did not identify these separately from the primary care visits. Rehabilitation services are also provided directly by the city of Oulu. However, these services were outsourced to a private provider and these services were not part of the NHI scheme so I did not have access to these data. NHI covered rehabilitation utilization was measured with a binary variable. This variable indicated whether a child utilized this service during a given quarter or not.

#### Psychiatric medication

Data on psychiatric medication purchases originated from the prescription register maintained by Social Insurance Institution of Finland. The register include all prescription purchases from pharmacies that were reimbursed by the NHI. The data include the date of prescribing and the ATC code. In the classification of psychiatric medication, I included depression medication, antipsychotics (excluding lithium), benzodiazepines, and benzodiazepine related drugs. See S1 File for the list of ATC codes. Psychiatric medication utilization was measured with a binary variable indicating whether a child utilized psychiatric medication in a given quarter or not.

#### Child protection service

Child protection information originated from the social service register of the city of Oulu. The data included variables for the month of the service and the type of social service. I included information that was labeled as “child protection.” These services included both in-home and out-of-home care. Child protection service utilization was measured with a binary variable indicating whether a child utilized this service in a given quarter or not.

#### Demographic information

Information on children’s age, sex, and place of residence was retrieved from the population register of the Social Insurance Institution of Finland. Age and municipality of residence were always recorded at the end of the statistical year.

### Ethics statement

The Finnish National Board on Research does not require an ethical review statement for studies using only register-based data [38]. The study used secondary data retrieved from registers, and no human subjects were contacted in the collection of the data. The data was fully pseudonymized before access was granted to the author. According to the General Data Protection Regulation of the EU (GDPR) [39] and the Finnish Data Protection Act [40], processing of personal data is permitted without informed consent for a task carried out in the public interest, such as scientific research. Because of the sensitive nature of the individual-level register data, the author does not have permission to make the data available. Legal restrictions prevent the public sharing of the data [40, 41]. The data were accessed through permissions from the city of Oulu (date of permission 20 Jan 2021), the Finnish Institute for Health and Welfare (date of permission 20 Jan 2021), and the Social Insurance Institution of Finland (date of permission 11 Feb 2021).

### Empirical method

Children with mental health conditions can differ from the rest of the population in many ways and their health care and other service utilization trajectories can follow different paths. As these children are not randomly allocated to treatment and control groups, direct comparison of children with and without these conditions likely yields biased estimates. To take into account this problem, I followed the previous literature [34, 35, 42, 43], and utilized a quasi-experimental research design. The literature has applied this research design to other settings but not to children’s mental health diagnoses.

The general idea is to overcome the challenge of selection bias and unobserved heterogeneity. The research design exploited the timing of the first mental health or behavioral disorder related diagnosis among those who were diagnosed during the sample period. Counterfactuals to the diagnosed group were drawn from those who received the same diagnosis three years later. Specifying the control group in this way increases the plausibility that the groups are similar.

**Fig 1:**
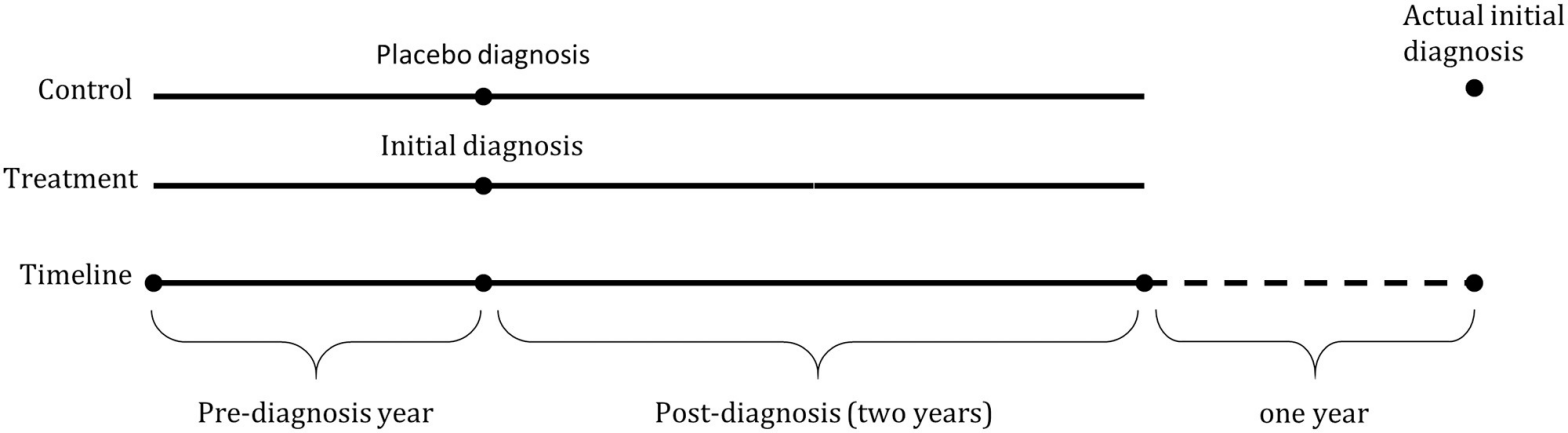
Illustration of the empirical setting. Solid line indicates the follow-up period used in the estimations. Data past the two-year post-diagnosis period were not analyzed in the estimations (dashed line).

Specifically, the treatment and control groups were formed from two waves: those who got the initial diagnosis in 2014 or 2015 (treatment) and those who got the initial diagnosis in 2017 or 2018 (control). For the control group, a “placebo” diagnosis was assigned to have been made three years earlier than the actual time of initial diagnosis. As such, the association of the conditions around the diagnosis was identified from the change in service utilization between the two groups over time. A three-year window was chosen to assure that the treatment and control groups remain comparable and to allow the outcomes to be tracked for a sufficient amount of time after diagnosis. Overall, service utilization was tracked for one year before and two years after diagnosis. Fig 1 illustrates the setting.

Additionally, I used propensity score matching (PSM) to further balance the treatment and the control groups with respect to observable characteristics. Specifically, the age (at the time of initial/placebo diagnosis), sex and the initial primary diagnosis (at three digit level) were used in the matching process. By matching on the initial diagnosis, a concern that the results could be driven by potential differences in the initial diagnoses between the groups is alleviated. Nearest neighbor matching algorithm with a caliper width equal to 0.2 of the standard deviation of the logit of the propensity score was used [44, 45]. Each child in the treatment group was assigned a unique matched counterpart from the control group. See S2 File for the fractions of the most common diagnoses for the two groups after matching. However, a concern may remain that those who received their initial diagnosis a few years later in their youth could still somehow systematically differ in unobservable characteristics (e.g. clinical severity). If these unobservable characteristics contributed to differential trends in the outcomes, the results could be affected.

The estimation framework was an event study (dynamic difference-in-differences) specification:

$$\begin{array}{cc} y_{it} & =\alpha +\beta {treatment}_{i}+\gamma_{t}+\lambda X_{it}+\sum_{\tau =-Q4,\tau \neq -Q1}^{Q7}\delta_{\tau }\times I_{\tau }\left(t=\tau \right)\\ & +\sum_{\tau =-Q4,\tau \neq -Q1}^{Q7}\theta_{\tau }\times I_{\tau }\left(t=\tau \right)\times treatment_{i}+\epsilon_{it}, \end{array}$$ (1)

where *y*_*it*_ denotes the outcome for child *i* at time *t*. Time was measured at quarterly intervals. *α* is a constant, *treatment*_*i*_ is a binary variable that denotes whether a child belonged to the treatment group, *γ*_*t*_ is a vector of time fixed effects, *X*_*it*_ contain controls for age fixed effects and sex, and *ϵ*_*it*_ is the error term. *I*_*τ*_(*t* = *τ*) are binary variables that indicate the quarter relative to the quarter of diagnosis. *τ* = *Q*0 is the quarter of diagnosis, with negative values indicating pre-diagnosis quarters and positive values post-diagnosis quarters. *τ* = −*Q*1 is the omitted quarter and thus the parameters are estimated relative to this period. *δ*_*τ*_ indicate the event study estimates for the control group. *θ*_*τ*_ are the parameters of interest and indicate the event study estimates for the treatment group. The parameter estimates *θ*_*τ*_ are recovered from the difference in the outcome between the treatment and control groups in the given quarter relative to the difference in their outcomes in the baseline quarter *τ* = −*Q*1. In other words, *θ*_*τ*_ indicate the differences in the outcome trajectories between the treatment and control groups.

The identification for *θ*_*τ*_ comes from the assumption that, absent the onset of a mental health condition, the outcomes for the treatment and control groups would run parallel. The plausibility of this assumption is increased by limiting the comparison to children who got the diagnosis. Also, because of the three-year time window, there is one-year gap year between the last quarter of the post-diagnosis tracking (*τ* = *Q*7) and the period of actual diagnosis in the control group. This mitigates the concern that potential changes in outcomes in the control group just before their actual diagnoses would bias the identification of the post-diagnosis parameters of the treatment group. However, if the outcomes in the control group were affected more than one year prior to the actual diagnosis, the parameter estimates for the post-diagnosis periods could still be biased. To verify this, I also plotted the trends in the outcomes for the control group to see if there were any notable changes more than one year prior to their actual diagnosis.

All outcomes were aggregated to a quarterly level. Poisson regression was used to estimate Eq 1 when the outcomes were count variables. These outcomes were the number of visits in public primary care, specialized health care, on-call duty, and private health care. For these outcomes, parameter estimates were reported as incidence rate ratios (IRR). IRRs greater than one indicate that the rate of visits increased more steeply in the treatment group than in the control group. IRRs less than one indicate that the rate of visits decreased more steeply in the treatment group than in the control group. For binary outcomes, the parameters were estimated using ordinary least squares (linear probability model). Outcomes measured with a binary variable were visits to a mental health professional, NHI covered rehabilitation, psychiatric mediation, and child protection service utilization. To take into account any within-child correlation in model errors *ϵ*_*it*_ across time, standard errors were clustered at the child level [46].

### Details on sample formation

See S3 File for a flowchart of the data processing and sample formation. The study sample was formed by identifying the earliest date of mental health diagnosis for each child. Those who had their earliest diagnosis in 2014 or 2015 were assigned to the treatment group. Those who had their earliest diagnosis in 2017 or 2018 were assigned to the control group. Because these children had to be followed for one year before and two years after their diagnosis (or “placebo” diagnosis), it was required that they had the city of Oulu as their place of residence during the whole three-year follow-up period. For example, those that received the initial diagnosis in 2014 (or “placebo” diagnosis in 2014), had to be residents of Oulu during 2013–2016. Another requirement was that the children in the treatment and control groups had to be aged 5–15 during the follow-up period.

The final sample consisted of 617 individual children in both the treatment group and the control group. Overall, the number of observations was 14,808 in the panel in the three-year follow-up period (12 quarters). This was the sample used in the regression estimations.

## Results

### Descriptive results

S4 File shows the service utilization separately for the treatment and control groups across the three-year follow-up period. Service utilization is measured with a binary variable. As described in the institutional setting, all children are entitled to child and school health care. Thus, in practice, as these visits are included in primary care visits, all children had visited primary care. The treatment group had a higher prevalence of specialized health care visits and on-call duty visits. Also, the prevalence of visiting a mental health professional, utilization of NHI covered rehabilitation, psychiatric medication, and child protection services was notably higher in the treatment group, possibly because of being earlier diagnosed in the follow-up period.

Table 1 shows descriptive statistics for the treatment and control groups across the three-year follow-up period. After matching, the mean age was similar between the groups. The fraction of females remained slightly higher in the control group. The mean number of health care visits per quarter was somewhat higher in the treatment group, albeit the standard deviations were large. Also, the quarterly probabilities for visiting a mental health professional, utilization of rehabilitative and child protection services, and being on psychiatric medication were slightly higher in the treatment group. The likely explanation for these differences is that, for the treatment group, the values were calculated from the pre- and post-diagnosis period, while for the control group, the values were calculated from the period before their actual diagnosis. Also, to receive a diagnosis in the first place, a health care professional needs to be visited.

**Table 1:** Descriptive statistics per quarter for the treatment and control groups across the three-year follow-up period.

	Treatment			Control		
	%	Mean	SD	%	Mean	SD
Male	54.3			50.1		
Female	45.7			49.9		
Age		8.90	2.41		8.97	2.45
Number of primary care visits		1.77	2.27		1.22	1.64
Number of specialized health care visits		0.29	0.98		0.14	0.63
Number of on call duty visits		0.07	0.31		0.05	0.25
Number of private care visits		0.10	0.41		0.11	0.48
Has visited a mental health professional		0.07	0.25		0.02	0.15
Has had NHI covered rehabilitation		0.04	0.20		0.02	0.13
Is on psychiatric medication		0.00	0.06		0.00	0.05
Has used child protection services		0.15	0.36		0.08	0.27
Total number of persons (N)	617			617		

Note: The mean values and standard deviations for the variables are calculated from the child-quarterly level (aggregated) data.

### Event study regressions

S5 File shows the mean values of the outcomes before and after the initial diagnosis. The results largely mirrored the event study regression results presented in this section. They also validate the identification assumption that the outcome trends in the control group were not yet affected by mental health and that leaving one extra year before the actual diagnosis was enough for purposes of the empirical design.

**Fig 2:**
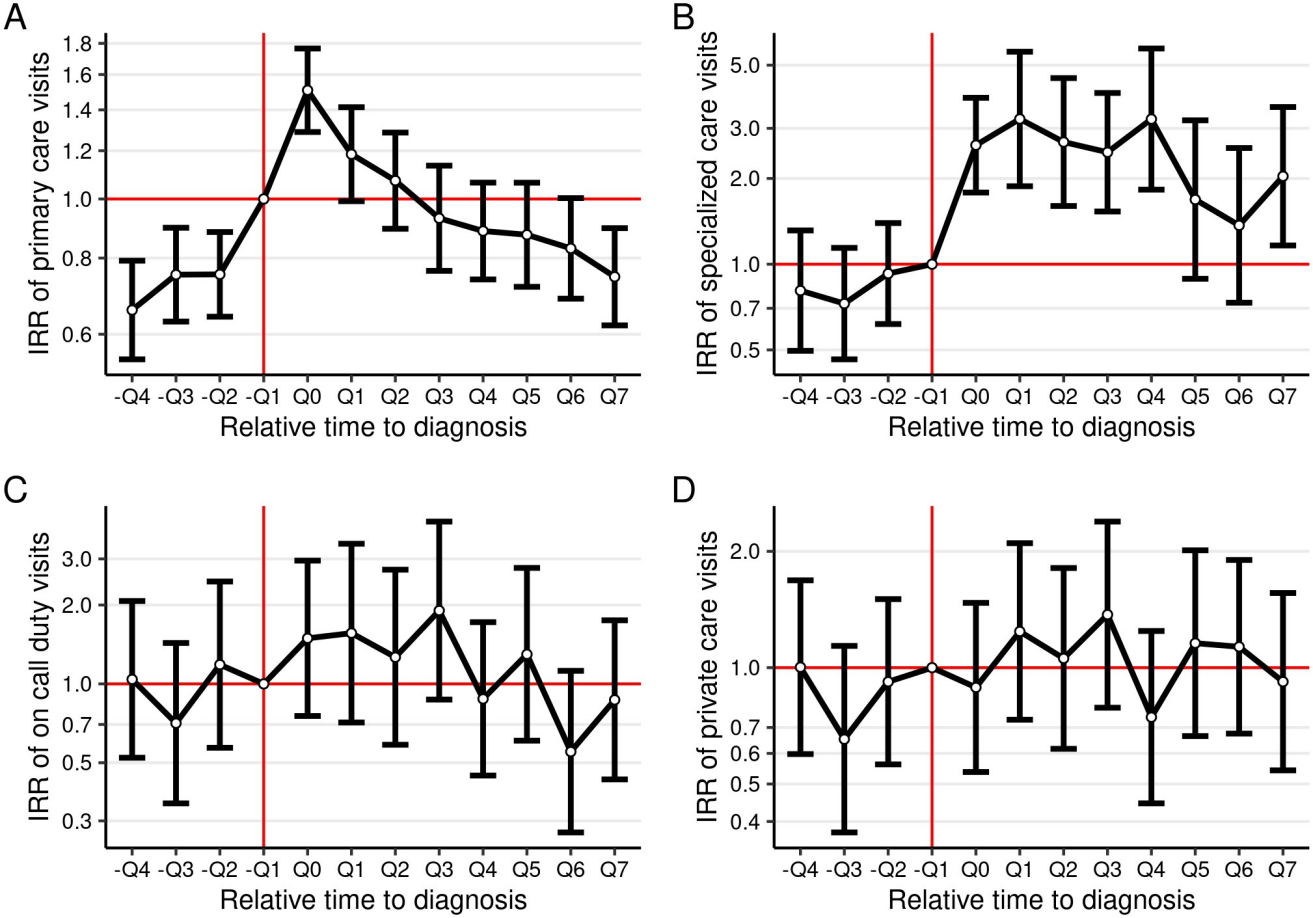
Event study regressions for number of visits. Y-axis shows the incidence rate ratio for the respective service. Y-axis is log-scaled. −*Q*1 is the omitted event dummy and coefficients are thus normalized with respect to this event-period. Controls used in the regressions include fixed effects for the common time trend, age, and sex.

Fig 2 plots the estimated parameters *θ*_*τ*_ from Eq 1 using Poisson regression (see S6 File for a table of the coefficients). The parameters were interpreted as the difference in the IRRs between the treatment and control groups in relation to quarter −*Q*1. The IRR of primary care visits showed an increasing trend in the pre-diagnosis period in the treatment group (Fig 2A). The IRR peaked during the quarter of diagnosis and then started decreasing in the following quarters. The IRR of specialized health care visits increased slightly but not statistically significantly in the pre-diagnosis period. During the quarter of diagnosis, the IRR of specialized health care visits was statistically significantly higher. The decreasing trend in the IRR in the post-diagnosis period was less steep than for primary care visits and remained statistically significantly higher. The estimated parameters for on-call duty visits and private care visits were more noisy and did not display any obvious and statistically significant patterns in the trend (Fig 2C and 2D).

**Fig 3:**
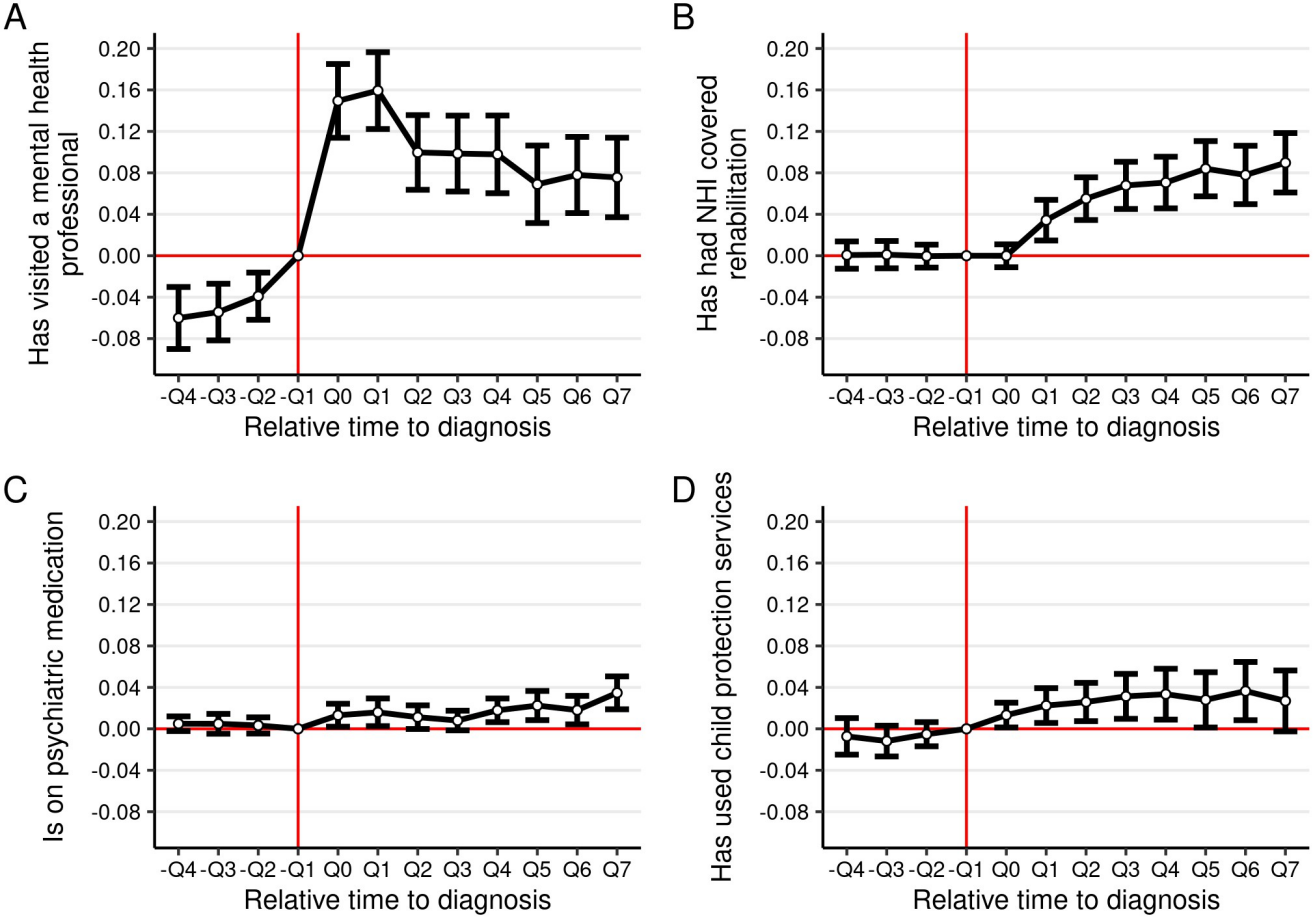
Event study regressions for service utilization. Y-axis shows the probability of utilization of the respective service. Service utilization is measured with a binary variable equaling one when the respective service was utilized and zero when not utilized. −*Q*1 is the omitted event dummy and coefficients are thus normalized with respect to this event-period. Controls used in the regressions include fixed effects for the common time trend, age, and sex.

Fig 3 plots the parameter estimates from the linear probability model (see S6 File for a table of the coefficients). In relation to quarter −*Q*1 and compared to the control group, the probability to visit a mental health professional increased by slightly over 15 percentage points during the period of diagnosis (Fig 3A). The probability for utilization of NHI covered rehabilitation increased significantly after the diagnosis, and peaked approximately a year after (Fig 3B). The probability of having psychiatric medication was statistically insignificant from zero in the pre-diagnosis periods (Fig 3C). In the post-diagnosis periods, the probability showed a statistically significant increasing trend. The point estimates for the probability of child protection service utilization increased statistically significantly in the post-diagnosis periods, by approximately 3–4 percentage points (Fig 3D). This is a 20% increase compared to the mean level of the outcome.

### More detailed descriptive comparison of service utilization

The results in the previous sections showed the trajectory of health care visits around the time of diagnosis. Here, I focused on a descriptive comparison of the service types and categories of the visits made to primary and specialized health care before and after diagnosis in the treatment group. Table 2 displays the fractions of these visits. For primary care visits, the fractions are calculated from the total number of primary care visits. For specialized care visits, the values are calculated from the total number of specialized care visits.

**Table 2:** Fractions of visits in different health care services types, before and after initial diagnosis.

	Pre-diagnosis (−*Q*4 – −*Q*1), %	Post-diagnosis (*Q*1 – *Q*4), %	Post-diagnosis (*Q*5 – *Q*7), %
Primary care visits			
Service type			
School health care	43.3	38.9	41.9
Outpatient care	22.9	14.7	16.7
Child health care	3.5	0.4	0.3
Mental health care	12.9	31.7	28.1
Other	17.4	14.3	13.1
Includes a mental health diagnosis	0.0	6.0	3.1
Specialized care visits			
Visit type			
With appointment	88.7	71.4	79.1
On call duty	6.6	4.3	5.2
Daytime hospital care	0.0	19.7	11.7
Other	4.7	4.7	4.0
Includes a mental health diagnosis	0.0	63.0	63.5

Notes: The table shows the fraction of visits in different service types separately for the periods before and after the initial diagnosis for the treatment group. For primary care visits, the fractions are calculated from the total number of primary care visits. For specialized care visits, the values are calculated from the total number of specialized care visits.

In primary care, the largest fraction, approximately 40% of the visits were labeled as school health care. However, the share of visits made to mental health care services increased from 13% to approximately 30% from pre-diagnosis to post-diagnosis year. In the specialized care visits, a majority, 89% in the pre-diagnosis year, and 71% in the post-diagnosis year, were made with an appointment. The largest increase came from daytime hospital care, which among others, includes intensified psychiatric treatment. In the post-diagnosis years, a bulk of the specialized care visits (63%) had a mental health diagnosis attached to them.

## Discussion

### Key results

This study analyzed the association of mental health and behavioral conditions with health care and service utilization during the year before the initial diagnosis and two years after the diagnosis. The number of primary care visits increased prior to the initial diagnosis. Specialized health care visits increased slightly but statistically insignificantly prior to the initial diagnosis. Also, the probability to visit a mental health care professional increased in the pre-diagnosis period.

Primary care visits peaked during the quarter of diagnosis and began to decrease after that, reverting back to the pre-diagnosis level two years after the diagnosis. In contrast, specialized health care visits remained higher in the post-diagnosis period. The probability to visit a mental health care professional followed a trajectory similar to specialized health care visits. The probabilities of utilizing NHI covered rehabilitation and psychiatric medication increased statistically significantly after the diagnosis. Also, the probability of child protection service utilization increased by approximately 20% compared to the mean level of the outcome.

In primary care, the fraction of visits to mental health care services increased from 13% to approximately 30% from pre-diagnosis to post-diagnosis year. In specialized health care, the largest increase came from daytime hospital care, in which, in the context of children, the treatment is typically mental health related. Additionally, the fraction of specialized health care visits that included a mental health diagnosis was 63% in the post-diagnosis period.

### Implications of the results

Studies that have analyzed specific conditions such as bipolar disorder [29, 30], schizophrenia [28], psychosis [27], and binge-eating disorder [31] have found an increased utilization of health care services preceding the diagnosis. While most of the studies did not focus on children and adolescents, the finding of increased health care visits before the initial diagnosis is in line with the previous results. Also, the inverted u-shape of primary care visits around the diagnosis is not unique in the literature [31, 32]. The similar trends seen in the utilization of specialized care and of mental health professionals are no surprise as many of these professionals work in specialized care.

Health care professionals working in primary care can play an important role in the treatment and referral of children to specialized care and mental health professionals [47]. Because children in Finland regularly visit child and school health care, the importance of these primary care services is emphasized in identifying mental health and behavioral conditions in children. Approximately 47% of the visits in primary care consisted of school and child health care during the pre-diagnosis year.

The results also highlight the importance of specialized care in the treatment following the diagnosis. Most of the treatment in specialized care was received in an outpatient setting. The decrease in primary care visits and the increase in specialized health care visits following the diagnosis indicate a transition of the treatment from primary care to specialized health care. Also, the finding that 63% of the specialized care visits had a mental health or behavioral diagnosis attached to them after the diagnosis indicates continuity of treatment in specialized care. Additionally, the increase in the share of daytime hospital care, which includes intensified psychiatric treatment, provides further evidence of this.

A confirmed diagnosis of a medical condition can be a precondition for receiving certain services. This likely explains the increased utilization of NHI covered rehabilitation and psychiatric medication after the diagnosis. Selection for rehabilitation is based on a physician’s referral and the final decision to grant rehabilitation is made by social insurance offices [48]. The rehabilitative measures can include, for example, rehabilitative psychotherapy and medical rehabilitation. It is likely that the more challenging and severe cases are referred into rehabilitative services.

The prevalence of psychiatric medication in children increases with age and is higher in adolescence [20, 21]. Typically, the prescribing of psychiatric medication in children is off-label based [20, 21]. Also, when health care resources are scarce, it could be that psychiatric medication is used as a substitute for other preferred treatments [49]. In the data, only around 7% of the children in the treatment group had used psychiatric medication. However, a year after the diagnosis, the probability of using psychiatric medication increased by 1–3 percentage points. Additionally, the use of psychiatric medication in children has been increasing over time [20, 21]. Thus, more research is needed on the reasons behind prescribing decisions and on the benefits of using psychiatric medication concurrently with other treatments.

Previous studies have found a strong association between foster care and mental health problems in children [23–25]. The results in this study showed that the probability of utilizing child protection services increased immediately during the quarter of the initial diagnosis. The increase could be related to parents’ need and demand for child service support to cope with their children’s mental health and behavioral conditions. In Finland, the less invasive in-home support measures are typically at first preferred over out-of-home service. Another indication of this result may be that health care professionals were able to identify the neglected children and their mental health conditions and make a referral to a social worker. And alternative explanation could be that, the mental health condition was assessed immediately after placing the child in child protection services. However, this would not explain the slightly increasing trend in the probability after the quarter of the initial diagnosis.

The special contribution of this study to the literature was the use of an empirical approach designed to tackle the problem of selection bias and unobserved heterogeneity in the population of interest. This approach enabled to plausibly study the association of the initial diagnosis with service utilization trajectories. In addition, this study used individual-level data gathered from several registers that enabled the analysis of not only health care utilization but also other service utilization such as child protection services. Also, the health care data included school and child health care, which was vital in the context of child population.

### Methodological considerations

A strength of this study was the combined register data gathered and merged from several different administrative registers that presented both health care and other service utilization in the population. Compared to survey data, which may suffer from response bias, register-based data is more reliable in terms of missing information and selection into data [50]. The data also enabled visits in primary and specialized care to be studied separately, which in turn offered a more comprehensive view on the health care utilization trajectories. Most importantly, the inclusion of child and school health care services in the outpatient health care data was crucial in terms of identifying health care utilization in the study population.

A strength in the empirical methodology was that it took into account selection bias by identifying the association of mental health separately from the difference in the timing of the initial diagnosis within the diagnosed population. The presumption was that children diagnosed later in the data were similar to children diagnosed earlier in the data. A naive comparison of the diagnosed population with the non-diagnosed population could yield biased estimates since the two populations are likely to differ in several unobservable confounders that cannot be controlled. These confounders, which could simultaneously affect mental health conditions, probability of diagnosis, and service utilization, may include individual health behaviors, the environment in which children are brought up, and comorbidities.

However, because the control group received the diagnosis later, a concern may still be that some systematic differences remained between the groups. For example, if a later received diagnosis correlates with clinical severity, and this clinical severity then affects the outcome trends, it may introduce bias to the parameters of interest. Matching with respect to the initial diagnosis alleviates some of this concern. However, within-diagnosis severity differences could still remain because of the potential later onset of the condition in the control group.

A drawback in the empirical methodology was the trade-off between the comparability of the treatment and control groups as well as the analysis horizon. The shorter the window between the early and later diagnosed is, the more similar the groups are likely to be. However, this directly also limits the length of the follow-up period after the event of interest. Thus, it is challenging to analyze very long-run associations with this method.

Even with the unique data set used in this study, a limitation was the sample size of the diagnosed population. This ruled out the possibility of performing a heterogeneity analysis by finer sub-classifications of the diagnoses. The results could differ between more severe and less severe diagnoses. Also, this study analyzed trajectories only around the initial diagnosis, and any following mental health and behavioral diagnoses were not studied. The initial diagnosis may precede more complex mental health conditions [29, 30], leading to differing service utilization patterns. It is also noteworthy that some children in the treatment group may have been diagnosed prior to the one year follow-back window if their mental health conditions had an episodic course. In these cases the initial diagnosis would be incorrectly identified, which could affect the results.

Furthermore, the study population was identified based on the diagnosis. Mental health and behavioral conditions can be underdiagnosed in children to some extent [50–52]. Thus, the service utilization of these children could not be studied here. Even with information on the service type and the mental health diagnoses included in the visits, mental health and physical health related visits could not be perfectly disentangled from each other. This could lead to, for example, under-reporting of mental health related visits. Some of the increase in primary and specialized care visits could also be due to physical health related visits. Unfortunately, the reasons for physical visits could not be determined from the data. These important questions should be the focus of further research.

## Conclusion

This study evaluated the association of mental health and behavioral conditions with health care and other service utilization before and after the initial diagnosis. Findings showed that primary care utilization increased sharply right before the initial diagnosis. Specialized health care utilization had a more minor increase prior to the diagnosis. Primary care utilization started to decrease towards the pre-diagnosis level shortly after, while specialized care utilization remained higher two years after the initial diagnosis. The probability to utilize mental health professionals, rehabilitation, psychiatric mediation, and child protection services increased significantly after the diagnosis.

The role of outpatient care in the detection and treatment of mental health and behavioral conditions is crucial in children and adolescents. Recognizing the trajectories of service utilization for children with these conditions is essential and could help policy makers in targeting resources within health care and social care to improve children’s health outcomes.

## Supporting information

S1 File Classification codes. (PDF)

S2 File Fractions of initial diagnosis codes for the treatment and control groups. (PDF)

S3 File Flowchart of data processing. (PDF)

S4 File Comparison of service utilization for the treatment and groups. (PDF)

S5 File Plots of outcome values before and after diagnosis. (PDF)

S6 File Event study regression tables. (PDF)
