# Supplementary material for: Association of mental health and behavioral disorders with health care and service utilization in children before and after diagnosis
Source: PLoS One. 2022 Nov 28;17(11):e0278198. doi: 10.1371/journal.pone.0278198 (PMC9704676; doi:10.1371/journal.pone.0278198)
Supplement: S1 File — (PDF) [file pone.0278198.s001.pdf]

# S1 File: Classification codes

## ATC-codes

- Antipsychotics: N05AA, N05AB, N05AC, N05AD, N05AF, N05AG N05AH, N05AL, N05AX
- Antidepressants: N06AA, N06AB, N06AF, N06AG, N06AX
- Benzodiazepine derivatives: N05BA, N05CD
- Zopiclone: N05CF01
- Zolpidem: N05CF02

## Mental health and behavioural diagnoses

- Register of the city of Oulu and the Register of Primary Health Care Visits
  - ICPC-2: diagnoses in the "P" category (Psychological)
- the Care Register of Health Care
  - ICD-10: diagnoses in the "F" category (mental and behavioural disorders)

## Mental health professionals

- Register of the city of Oulu and the Register of Primary Health Care Visits
  - 24451 and (psychologist), 24452 (psychotherapist), 24454 and 32262 (occupational therapist)
- the Care Register of Health Care
  - 70 (psychiatrist), 70X and 74 (adolescent psychiatrist), 75 and 75X (child psychiatrist)
