# Supplementary material for: Association of mental health and behavioral disorders with health care and service utilization in children before and after diagnosis
Source: PLoS One. 2022 Nov 28;17(11):e0278198. doi: 10.1371/journal.pone.0278198 (PMC9704676; doi:10.1371/journal.pone.0278198)
Supplement: S2 File — (PDF) [file pone.0278198.s002.pdf]

S2 File: Fractions of initial diagnosis codes for the matched treatment and control groups

Table S1: **Fractions of the 10 most frequent initial diagnoses for the matched treatment and control groups.**

Diagnosis code	Name	Treatment	Control
P22	Child behaviour symptom/complaint	0.14	0.13
F90	Attention-deficit hyperactivity disorders	0.07	0.08
P29	Bedwetting/enuresis	0.06	0.08
P01	Feeling anxious/nervous/tense	0.06	0.06
F98	Other behavioral and emotional disorders with onset usually occurring in childhood and adolescence	0.05	0.06
F45	Somatoform disorders	0.05	0.06
F93	Emotional disorders with onset specific to childhood	0.05	0.04
P06	Sleep disturbance	0.04	0.06
F40	Phobic anxiety disorders	0.04	0.03
F81	Specific developmental disorders of scholastic skills	0.03	0.03

Notes: Treatment and control groups are matched using propensity score matching. See main text for more information. Diagnosis codes are either in ICD10 (begins with "F") or ICPC-2 (begins with "P") coding system.
