# Supplementary figures and images for: Association of mental health and behavioral disorders with health care and service utilization in children before and after diagnosis
Source: PLoS One. 2022 Nov 28;17(11):e0278198. doi: 10.1371/journal.pone.0278198 (PMC9704676; doi:10.1371/journal.pone.0278198)

### S3 File: Flowchart of data processing.

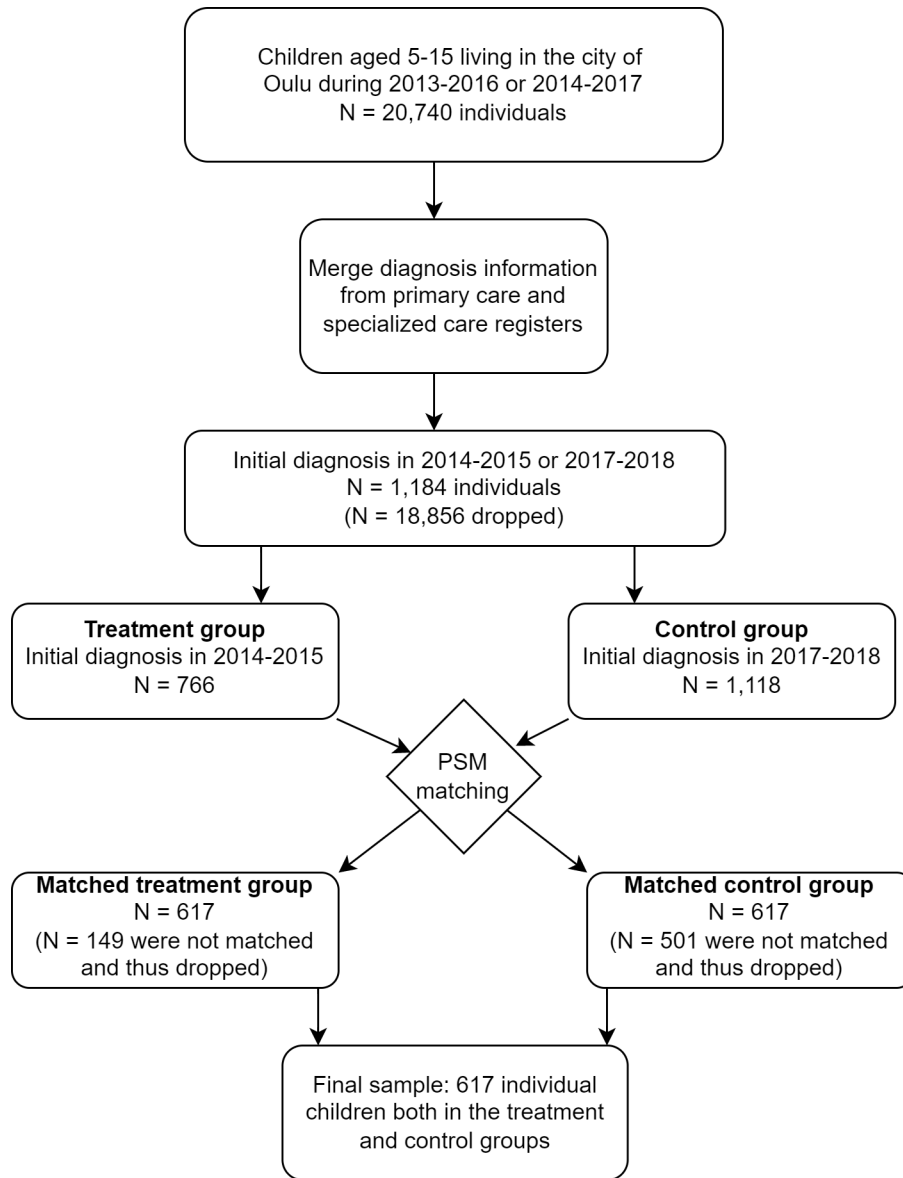

Figure S1: **Flowchart of data processing.**

Supplement: S3 File — (PDF) [file pone.0278198.s003.pdf]
