# Supplementary material for: Association of mental health and behavioral disorders with health care and service utilization in children before and after diagnosis
Source: PLoS One. 2022 Nov 28;17(11):e0278198. doi: 10.1371/journal.pone.0278198 (PMC9704676; doi:10.1371/journal.pone.0278198)
Supplement: S4 File — (PDF) [file pone.0278198.s004.pdf]

S4 File: Comparison of service utilization for the treatment and control groups.

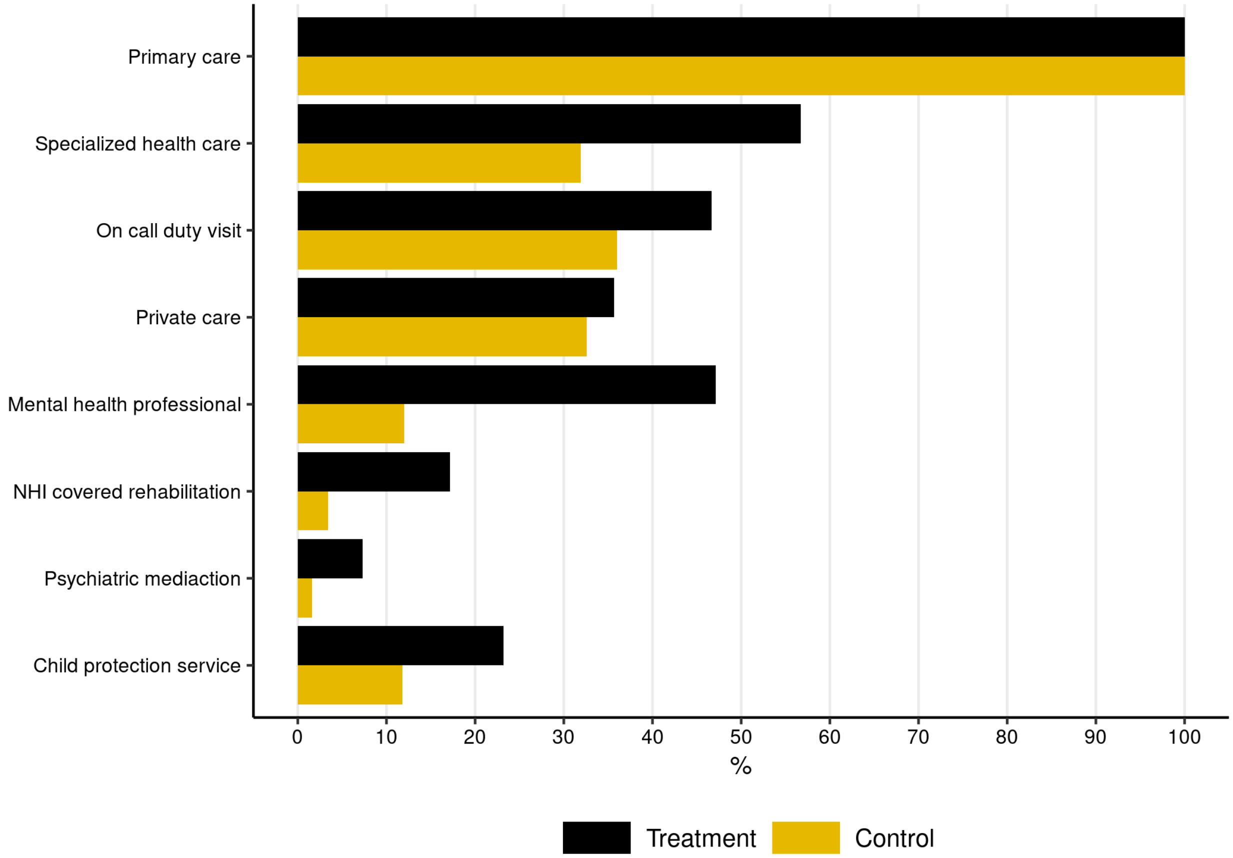

Figure S1: **Comparison of service utilization for the treatment and control groups.** The figure displays service utilization across the three-year follow-up period. Service utilization is measured with a binary variable, i.e. whether service was utilized at least once during the follow-up period.
