# Supplementary material for: Association of mental health and behavioral disorders with health care and service utilization in children before and after diagnosis
Source: PLoS One. 2022 Nov 28;17(11):e0278198. doi: 10.1371/journal.pone.0278198 (PMC9704676; doi:10.1371/journal.pone.0278198)
Supplement: S5 File — (PDF) [file pone.0278198.s005.pdf]

S5 File: Plots of outcome values before and after diagnosis.

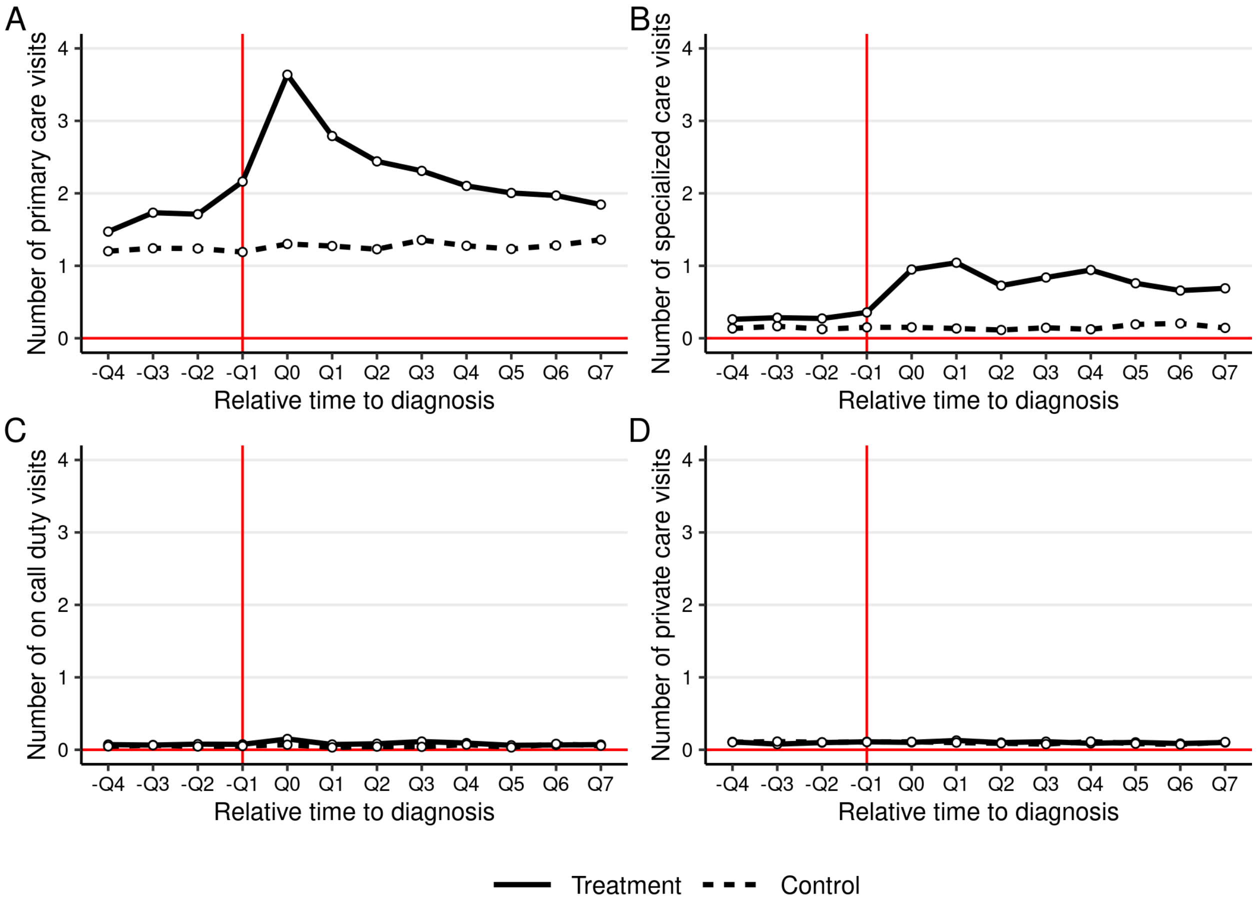

Figure S5A: **Number of visits before and after initial diagnosis.** Y-axis shows the mean number of visits to the respective service.

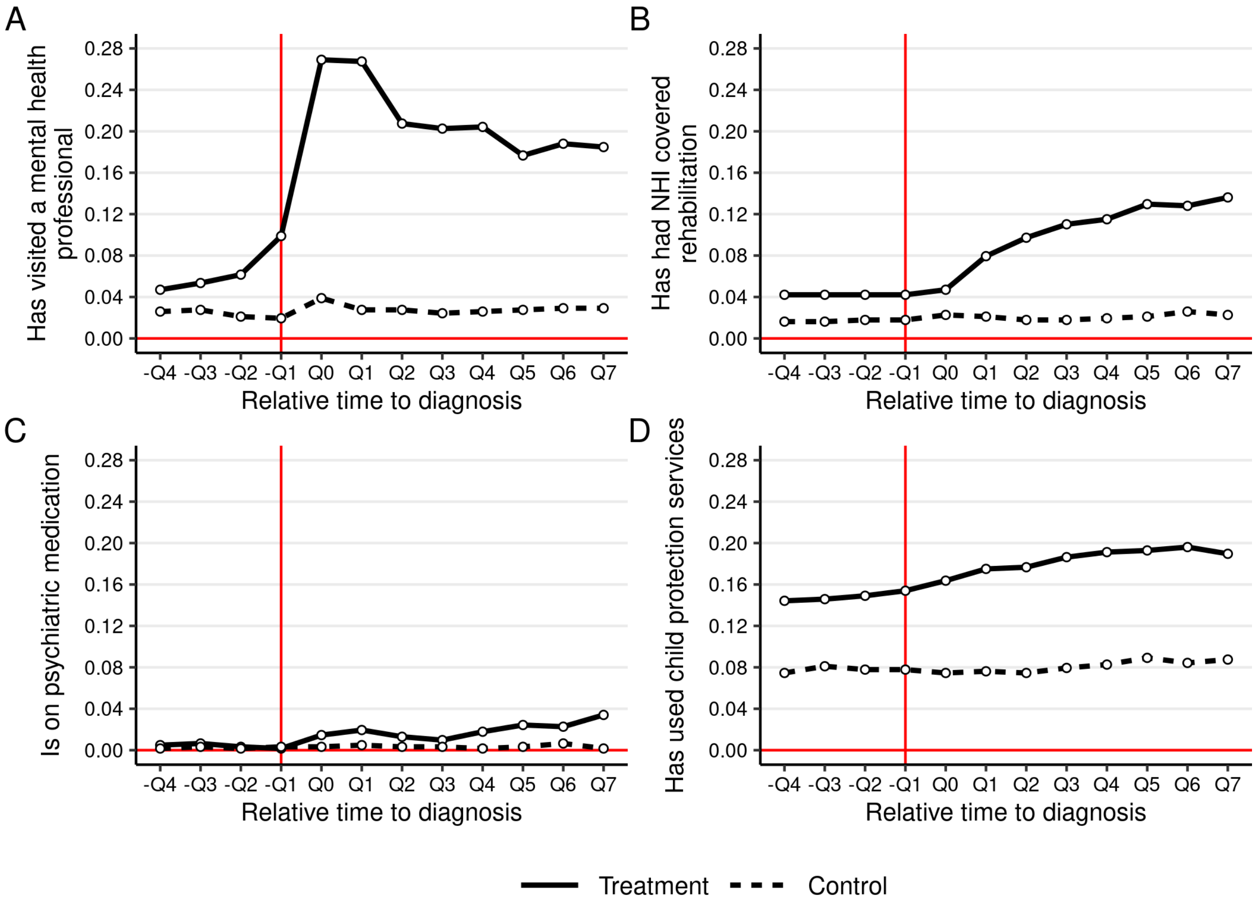

Figure S5B: **Service utilization before and after initial diagnosis.** Y-axis shows the mean probability of service utilization, measured with a binary variable equaling one when the respective service was utilized and zero when not utilized.
