# Supplementary material for: Association of mental health and behavioral disorders with health care and service utilization in children before and after diagnosis
Source: PLoS One. 2022 Nov 28;17(11):e0278198. doi: 10.1371/journal.pone.0278198 (PMC9704676; doi:10.1371/journal.pone.0278198)
Supplement: S6 File — (PDF) [file pone.0278198.s006.pdf]

S6 File: Event study regression tables

Table S1: **Poisson event study regression incidence rate ratios and 95% confidence intervals.**

	Primary care visits		Specialized care visits		On call duty visits		Private care visits	
Variable	IRR	CI	IRR	CI	IRR	CI	IRR	CI
$-Q4$	0.66***	(0.55, 0.79)	0.81	(0.50, 1.31)	1.04	(0.52, 2.07)	1.00	(0.60, 1.68)
$-Q3$	0.75***	(0.63, 0.90)	0.73	(0.46, 1.14)	0.71	(0.35, 1.43)	0.65	(0.37, 1.14)
$-Q2$	0.75***	(0.64, 0.88)	0.93	(0.62, 1.39)	1.18	(0.57, 2.46)	0.92	(0.56, 1.50)
$-Q1$	1.00		1.00		1.00		1.00	
$Q0$	1.51***	(1.29, 1.77)	2.62***	(1.79, 3.84)	1.49	(0.75, 2.95)	0.89	(0.54, 1.47)
$Q1$	1.18*	(0.99, 1.41)	3.24***	(1.88, 5.57)	1.56	(0.71, 3.43)	1.24	(0.73, 2.10)
$Q2$	1.07	(0.89, 1.29)	2.69***	(1.60, 4.50)	1.26	(0.59, 2.73)	1.06	(0.62, 1.81)
$Q3$	0.93	(0.76, 1.13)	2.47***	(1.53, 3.99)	1.90	(0.87, 4.16)	1.37	(0.79, 2.39)
$Q4$	0.89	(0.74, 1.06)	3.23***	(1.83, 5.72)	0.88	(0.45, 1.72)	0.74	(0.45, 1.24)
$Q5$	0.87	(0.72, 1.06)	1.69	(0.89, 3.20)	1.30	(0.61, 2.77)	1.16	(0.67, 2.01)
$Q6$	0.83*	(0.69, 1.00)	1.37	(0.73, 2.56)	0.55	(0.27, 1.12)	1.13	(0.68, 1.90)
$Q7$	0.75***	(0.62, 0.90)	2.04**	(1.16, 3.56)	0.87	(0.43, 1.75)	0.92	(0.54, 1.56)

Notes: $-Q1$ is the omitted event dummy and coefficients are thus normalized with respect to this event-period. Controls used in the regressions include fixed effects for the common time trend, age, and sex.
* $p < 0.1$, ** $p < 0.05$, *** $p < 0.01$

Table S2: **Linear probability model event study regression coefficients and 95% confidence intervals.**

	Mental health professional		Rehabilitation		Psychiatric medication		Child protection services	
Variable	Estimate	CI	Estimate	CI	Estimate	CI	Estimate	CI
$-Q4$	-0.06***	(-0.09, -0.03)	0.00	(-0.01, 0.01)	0.00	(0.00, 0.01)	-0.01	(-0.02, 0.01)
$-Q3$	-0.05***	(-0.08, -0.03)	0.00	(-0.01, 0.01)	0.00	(0.00, 0.01)	-0.01	(-0.03, 0.00)
$-Q2$	-0.04***	(-0.06, -0.02)	0.00	(-0.01, 0.01)	0.00	(0.00, 0.01)	-0.01	(-0.02, 0.01)
$-Q1$	0.00		0.00		0.00		0.00	
$Q0$	0.15***	(0.11, 0.19)	0.00	(-0.01, 0.01)	0.01**	(0.00, 0.02)	0.01**	(0.00, 0.03)
$Q1$	0.16***	(0.12, 0.20)	0.03***	(0.01, 0.05)	0.02**	(0.00, 0.03)	0.02***	(0.01, 0.04)
$Q2$	0.10***	(0.06, 0.14)	0.06***	(0.03, 0.08)	0.01*	(0.00, 0.02)	0.03***	(0.01, 0.04)
$Q3$	0.10***	(0.06, 0.14)	0.07***	(0.05, 0.09)	0.01*	(0.00, 0.02)	0.03***	(0.01, 0.05)
$Q4$	0.10***	(0.06, 0.14)	0.07***	(0.05, 0.10)	0.02***	(0.01, 0.03)	0.03***	(0.01, 0.06)
$Q5$	0.07***	(0.03, 0.11)	0.08***	(0.06, 0.11)	0.02***	(0.01, 0.04)	0.03**	(0.00, 0.05)
$Q6$	0.08***	(0.04, 0.11)	0.08***	(0.05, 0.11)	0.02**	(0.00, 0.03)	0.04**	(0.01, 0.06)
$Q7$	0.08***	(0.04, 0.11)	0.09***	(0.06, 0.12)	0.03***	(0.02, 0.05)	0.03*	(0.00, 0.06)

Notes: $-Q1$ is the omitted event dummy and coefficients are thus normalized with respect to this event-period. Controls used in the regressions include fixed effects for the common time trend, age, and sex.
* $p < 0.1$, ** $p < 0.05$, *** $p < 0.01$
